# A review of knowledge, attitudes and practices regarding mosquitoes and mosquito-borne infectious diseases in nonendemic regions

**DOI:** 10.3389/fpubh.2023.1239874

**Published:** 2023-12-08

**Authors:** Pénélope Duval, Christina Aschan-Leygonie, Claire Valiente Moro

**Affiliations:** ^1^Universite Claude Bernard Lyon 1, Laboratoire d’Ecologie Microbienne, UMR CNRS 5557, UMR INRAE 1418, VetAgro Sup, Villeurbanne, France; ^2^UMR 5600 Environnement Ville Société, University of Lyon, Université Lumière Lyon 2, Lyon, France

**Keywords:** knowledge, attitude, practice, mosquitoes, mosquito-borne infectious diseases (MBIDs), nonendemic regions, preventive methods

## Abstract

Mosquito-borne infectious diseases (MBIDs) present significant public health risks within tropical and subtropical regions. However, the rapid spread of MBIDs from these areas to temperate regions increase the risk of their emergence in nonendemic regions, i.e., regions where diseases are still sporadic and not sustained in the population. Raising awareness about preventive measures and protective behaviors is of primary importance to face the risks of vector-borne diseases. In this context, the number of studies on knowledge, attitude, and practice (KAP) about mosquitoes and MBIDs has grown rapidly in response to the need to identify knowledge and practices in nonendemic countries to fight mosquito proliferation. Building upon the recent developments in this field, we conducted the first-ever literature review to examine KAP studies conducted in nonendemic regions. Our aim was to identify the community’s knowledge and attitudes that shape practices concerning the prevention of MBIDs. We used specific keywords regarding the scope of this review and then selected studies that were performed in nonendemic regions for MBIDs, including regions located in European countries, the USA or Asia. We identified 32 KAP studies, the oldest from 2003. The findings in the reviewed studies show that survey participants generally possessed a rather good understanding of mosquito breeding sites. However, there were notable variations in knowledge and perception of MBIDs, primarily linked to the geographic location of the survey and the prevalence of infectious outbreaks related to mosquito transmission. These findings highlight the significant influence of knowledge and awareness in fostering effective mosquito control practices. Moreover, socioeconomic status, particularly educational attainment, and respondents’ gender emerged as key determinants in explaining the variability of appropriate practices. The survey results thus show the crucial role of knowledge, emphasizing the need for widespread awareness and information campaigns, encompassing both appropriate practices and efficient mosquito control methods. Understanding the interaction between these factors could provide good guidelines for implementing awareness plans and ultimately motivate the population to actively fight against mosquito proliferation and MBIDs development.

## 1 Introduction

Global climate change has intensified concerns regarding mosquito-borne infectious diseases (MBIDs) due to the expanding global distribution of arboviruses transmitted by mosquitoes in recent years (1). Nonendemic regions, referring to areas where diseases remain sporadic and are not sustained within the population, are increasingly grappling with this problem, as seen in various European countries and the United States, for instance. The expansion of mosquito vector species in these areas is further compounded by poor mosquito prevention and control practices (2). MBIDs are transmitted to people through the bite of an infected female mosquito, which lays its eggs on water surfaces in various habitats, such as salt marshes, lakes or ponds, polluted water retention systems, or any other location where water accumulates. While all mosquitoes require standing water to reproduce, different mosquito species thrive in different habitats, including temporary water habitats or water sources that remain for long periods (3). For instance, some *Aedes* mosquito species have evolved to lay their eggs in artificial containers. This is why urban areas are becaming suitable environments for the development of these species by providing mosquitoes with a multitude of man-made breeding sites, such as flowerpots, empty containers, and rain gutters, leading to their rapid colonization in cities (4).

Considering the absence of prophylactic measures or vaccines for the majority of MBIDs, effective control of the mosquito vector stands as a crucial step in curtailing the proliferation of both mosquitoes and these diseases (5). Vector control measures rely on the use of different individual or collective practices. Among them, chemical insecticides are widely used. However, their adverse impacts on nontarget species and their secondary effects due to resistance in mosquito populations have made chemical insecticides a controversial social issue (6). On the other hand, environmental measures such as draining standing water, proper disposal of empty containers or the use of bed nets on standing water containers are considered sustainable methods to control the spread of *Aedes* and *Anopheles* mosquitoes in urban areas (7). To be effective, these methods must be used in conjunction with other control methods and involve the local community. Additionally, public health risk awareness, ovitrap surveillance, and door-to-door control measures contribute to reducing the risks of spreading MBIDs (8). The choice between different existing and emerging vector control methods or combinations of techniques must be driven by the epidemiological, environmental, and socioeconomic context (9). One of the most significant explanations for the invasion and proliferation of mosquito vectors in urban areas is the practices of city residents (10). Knowledge, attitude and practices (KAP) questionnaires are frequently used to assess which parameters influence good or bad practices as well as knowledge and attitudes regarding mosquitoes and MBIDs. These surveys focus mainly on the mosquito life cycle, mosquito ecology, mosquito identification, biting times, control methods, and transmitted diseases. These surveys are essential to identifying inappropriate population behaviors and targeting the lack of awareness of a disease and/or vector as well as finding efficient strategies that involve the community in vector control. Given the importance of this issue, a review of studies based on KAP surveys can provide decision-makers with new information to better manage mosquito spreading and health risks. This study is the first to synthesize current knowledge on 32 published KAP studies conducted in nonendemic regions.

## 2 Materials and methods

Searches were carried out on Google Scholar in February and March 2023 from peer-reviewed journal articles and reviews written in English, without period restriction. We first used a list of specific keywords that matched with the scope of our study. This first process of selection allowed us to identify 903 articles. Then, we kept articles that only concerned studies that were performed in countries that are considered as nonendemic regions (or at least partially, like Vietnam) for MBIDs, i.e., regions where diseases are still sporadic and not sustained in the population. It includes studies conducted across various European countries, such as regions in France, Spain, Germany, Greece, and Italy, as well as a diverse array of states in the USA, including California, Florida, Louisiana, Utah, Alabama, and Arizona. Additionally, it encompasses research from regions in Asian countries such as Korea, Vietnam, Bangladesh, India, Malaysia, and Turkey. This second selection process leaded to the selection of 76 articles. Finally, after removing duplicates or articles that did not match our inclusion criteria (no KAP survey, type of article), our search yielded a total of 32 studies that matched our inclusion criteria (Table 1). These studies differed according to several factors such as the country/city, the mosquito species, MBIDs and targeted populations (Table 2).

**Table 1:** Selection criteria used for studies considered for the review.

Key words	Number of articles	Filter 1: studies in nonendemic countries	Filter 2: study types
attitude *Aedes*	13	1	1
attitude *Culex*	0	0	0
attitude *Anopheles*	0	0	0
attitude mosquito	45	1	1
attitudes mosquito	26	8	3
education mosquito	32	4	0
KAP mosquito	3	0	0
KAP mosquitoes	3	0	0
survey mosquito	344	8	8
knowledge mosquito	157	20	14
perception A*edes*	9	2	2
perception *Anopheles*	3	0	0
perception *Culex*	1	0	0
perception mosquito	35	3	1
perceptions mosquito	42	15	13
practice mosquito	42	0	0
practices mosquito	111	9	1
protective behaviors mosquito	2	2	2
questionnaire mosquito	6	2	1
socioeconomic mosquito	21	2	1
additional records identified through other sources	8	8	8
Total with duplicates	903	85	56
Total without duplicates	-	-	32

**Table 2:** Characteristics of selected studies (n = 32).

Continent	Country	City	Mosquito species and/or MBIDs	Participants	Women (%)	Men (%)	Focus group	References	
America	USA	NA	*Aedes* sp. */* Zika	59	100	0	Gynecology patients	[25]	Curry et al. (2017)
		Key West/Tucson	*Aedes aegypti*	775	49	51	Randomly selected participants	[53]	Haenchen et al. (2016)
		Provo (Utah)	Malaria, Dengue, Zika, Chikungunya and West Nile	1,043	68	32	Participants that traveled outside USA	[63]	Omodior et al. (2018)
		Louisiana	*Culex* sp. and *Aedes* sp.	260	64	30	Randomly selected participants	[37]	Moise et al. (2022)
		six states in the Northeast	*Culex* sp.	922	44	56	Randomly selected participants	[38]	Herrington et al. (2003)
		St. Johns County (Florida)	All mosquito species	24	NA	NA	Randomly selected participants in neighborhoods	[14]	Davidson et al. (2016)
		Region of southwestern Virginia	*Culex* sp. and *Aedes* sp.	188	NA	NA	Randomly selected participants	[34]	Butterwoth et al. (2010)
		Tennesse/Texas	La Crosse encephalitis	70	69	30	Randomly selected participants	[33]	Barnes et al. (2022)
		Miami	*Aedes* sp.	44	25	75	Outdoor contruction workers	[42]	Moore et al. (2017)
		Alabama	Malaria, Dengue, Zika, Chikungunya and West Nile	126	NA	NA	Randomly selected participants	[15]	Morse et al. (2019)
		NewYork	*Culex* sp.	97	68	32	Randomly selected participants in two neighborhoods (residential area/suburban area)	[12]	Tuiten et al. (2009)
		Tucson (Arizona)	*Aedes aegypti*	355	NA	NA	Randomly selected participants	[48]	Walker et al. (2018)
		Washington	*Culex* sp. and *Aedes* sp.	184	NA	NA	Randomly selected participants in five neighborhoods	[41]	Dowling et al. (2013)
		San Antonio (South Texas)	*Aede*s sp.	31	90	65	Randomly selected participants in five neighborhoods	[17]	Bohmann et al. (2022)
		Three cities in North Carolina	All mosquito species	415	49	50	Randomly selected participants	[43]	Richards et al. (2017)
		Florida	*Aede*s sp.	413	NA	NA	Physicians	[24]	Doblecki-Lewis et al. (2016)
Asia	Korea	Seoul, Gyeonggi-do, and Incheon	*Aedes* sp.	249	88	12	Nursing students	[46]	Choi et al. (2018)
	Vietnam	Hanoi	Dengue and Japanese encephalitis	513	30	70	Participants in 3 livestock households and 3 non-livestock households	[16]	Nguyen-Tien et al. (2021)
Asia/Europe	Bangladesh, India, Malaysia, and Turkey	NA	*Aede*s sp.	223	53	47	Physicians	[30]	Koonisetty et al. (2021)
Europe	Germany	NA	*Anopheles plumbeus*	118	31	64	Persons involved in citizen sciences project	[19]	Heym et al. (2017)
	Spain	Valldoreix	*Aedes albopictus*	309	60	40	Users of the Health Care Center	[11]	Curcó et al. (2008)
		Sant Cugat	*Aedes albopictus*	820	65	35	Randomly selected participants	[13]	Abramides et al. (2013)
	France	Lyon	*Aedes albopictus*	222	51	49	Community gardeners	[18]	Duval et al. (2022)
		South of France	*Aedes albopictus*	1,506	NA	NA	Randomly selected participants	[27]	Raude et al. (2012)
		Languedoc-Roussillon, Provence-Alpes-Côte d’Azur; Corsica	*Aedes albopictus*	574	55	45	Randomly selected participants	[31]	Constant et al. (2020)
		Camargue, petit camargue, Rhône alpes	All mosquito species	639	NA	NA	Randomly selected participants	[6]	Claeys et al. (2009)
	Greece	Athens	*Aedes* sp.	303	56	44	Participants in camp for migrants and in neighboring residential areas and urban areas	[23]	Kolimenakis et al. (2022)
		All administrative regions	*Aedes* sp.	573	100	0	Pregnant women	[28]	Mouchtouri et al. (2017)
		Vravrona	*Aedes albopictus*	40	50	48	Randomly selected participants	[22]	Stefopoulou et al. (2021)
	Italia	Lazio region	*Aedes albopictus*	1,579	55	45	Native populations and two resident communities originating from India	[21]	Caputo et al. (2020)
	Montenegro	Beciˇ ci,	*Aedes japonicus*	48	29	69	European participants of EMCA conference	[29]	Ibañez-Justicia et al. (2019)
	UK	NA	*Anopheles* sp.	125	60	44	Participants who plan to visit a malaria-endemic area	[45]	Goodyer et al. (2014)

## 3 Results

### 3.1 Knowledge, attitude and practices regarding mosquito development and associated health risks

#### 3.1.1 Knowledge of larval breeding sites and MBIDs

The reviewed studies revealed that a major part of the participants in the surveys had very good knowledge of the role of standing water as potential mosquito breeding sites (11–18). For instance, results from campaign prospecting door-to-door visits and interviews in Sant Cugat, Spain, showed that 84.9% of the surveyed persons had good knowledge about *Ae. albopictus* aquatic habitats (13). Similarly, Tuiten et al. (12) found that 99% of the respondents in New York (United States) knew that *Culex* mosquitoes breed in standing water (12). Morse et al. (15) obtained similar results in a survey in Alabama (United States) where 98% of the inhabitants identified standing water as sites that are suitable for mosquito breeding (15). In Lyon (France) a survey carried out amongst people participating in community gardens showed that 94.7% of the respondents knew that the tiger mosquito (*Aedes albopictus*) breed in small or medium standing water (18). Thus, overall, respondents in KAP studies recognized standing water containers as potential egg-laying sites.

However, these studies also showed that a significant proportion of the surveyed persons had only a partial knowledge of the type of containers used as breeding sites, or identified other locations that are not suitable as breeding sites (15, 18–23). For instance, in Lyon, 81.6% of the respondents believed that tiger mosquitoes breed in vegetation that in fact is suitable resting sites for adults, but cannot serve for larval development (18). Morse et al. (15) showed that empty containers or rain gutters were not associated with potential breeding sites even though they represent highly suitable larval habitats for mosquito colonization (empty containers and rain gutters were not identified by 49 and 39%, respectively). Caputo et al. (21) also revealed low knowledge about the breeding site characteristics of the Asian tiger mosquito *Aedes albopictus*; only 21.3% of the respondents had correct answers regarding knowledge on places where tiger mosquitoes lay eggs and larvae develop. In Greece, a minority of respondents was able to identify correctly the place were mosquitoes lay their eggs and larvae grow (20, 22, 23). Similar observations were made from a German study where only 11.6% of respondents identified tree holes as potential water-holding larval habitats in their gardens (19).

Although these KAP studies were conducted in nonendemic areas where MBIDs are still sporadic, they reveal different levels of knowledge about these infectious diseases according to places and targeted population in the surveys. Firstly, a good knowledge was evidenced in surveys performed in the South of the United States, where local transmissions occur, i.e., mosquitoes in the area have been infected with the virus and are spreading it to people (12, 14, 15, 17, 24–26). For instance, Bohmann et al. (17) demonstrated that survey participants in South Texas had a high level of knowledge on different diseases transmitted by mosquitoes: 100, 98 and 79% of respondents having previously heard of West Nile virus (WNV), Zika virus (ZIKV) and Dengue virus (DENV), respectively (17). By contrast, in Europe, MBIDs are still sporadic and epidemic events are mostly dependent on imported cases (i.e., when diseases are contracted abroad). Several studies found that knowledge of diseases transmitted by mosquitoes was limited and that people are generally unaware of the health risks (21, 22, 27–30). Mouchtouri et al. (28) found that only 9.6% of the interviewed Greek women answered correctly at all questions concerning Zika virus (28). Constant et al. (31) found that the use of entomological surveillance of the main mosquito arbovirus vectors in anticipation of the emergence risk of MBIDs generates a significant increase in the population’s knowledge of these diseases. Only 51.1% of the respondents residing in French Mediterranean regions knew that tiger mosquitoes could transmit chikungunya in 2012 compared to 88.3% in 2014 (31). In metropolitan France, health risks related to mosquito proliferation have increased significantly since 2011 when only 9 departments, out of 96, were colonized by the tiger mosquito compared to 81 in 2023 (84% of the departments). During the same period, the total number of imported cases of dengue, zika and chikungunya increased from 15 in 2013 to approximately 1,126 cases in 2023 (of which 1,099 cases of dengue). This evolution is probably increasing more anxiety in the population toward these emerging diseases (32). For example, Duval et al. (18) found that a total of 88.5% of community gardeners in Lyon, France, knew that mosquitoes could infect humans and animals (18). In comparison, Caputo et al. (21) showed that 49% of the Italian residents knew that mosquitoes can transmit diseases but did not know which ones. Only 1% had already heard about dengue, chikungunya or yellow fever (21). Similarly, Stefopoulou et al. (22) highlighted that only one respondent correctly identified the MBIDs among a list of 9 diseases (22). In 2018, 67.3% of the residents in Attica region in Greece where not able to list diseases that mosquitoes can transmit. Malaria was the most frequently answered response, followed by west nile fever and dengue. It is clear that some viruses are less known by the populations in certain regions (22). For instance, a large part of the population did not know about la crosse encephalitis (LAC) virus while this is the most medically significant California encephalitis virus in the USA (33, 34). Only 16.3% of respondents were conscious of the existence of LAC (34).

Travelers and migrants coming from endemic countries could have an impact on the knowledge and practices regarding mosquitoes in nonendemic countries. For instance, in Italy, a questionnaire-based study was conducted to compare the KAP of native populations and two communities originating from the Indian subcontinent where MBIDs are a major public health concern. The results showed higher knowledge and concern about *Aedes*-transmitted diseases in Indian communities associated with a lower level of concern of nuisance caused by mosquitoes (21). This suggests that people who have been exposed to MBIDs are better prepared to face epidemics. More generally, it appears that populations from endemic-mosquito areas have a higher awareness of MBIDs; therefore, it could be expected that migration to nonendemic countries could improve local population knowledge (35, 36). To a lesser extent, temporary visitors such as tourists may also affect knowledge of local populations. Interestingly, pregnant women from Greece who traveled abroad (not exclusively in endemic countries) had better knowledge about the risks of MBIDs than women who had not traveled abroad (OR = 10.47, *p* value = 0.04) (28). The authors suggested that this difference could be explained by a higher awareness among individuals who have traveled to endemic areas.

To improve knowledge about mosquitoes and MBIDs, educational campaigns are implemented by public services. Among different information sources, participants of KAP surveys are generally informed by mass media (TV, radio, websites and newspaper) (12, 18, 23, 25, 28, 37). Tuiten et al. (12) reported that 70% (n = 97) of the respondents mentioned that newspaper was the main information source about WNV followed by television (36%), radio (26%) and websites (20%) (12). Similar observations were made in the Lyon study in which 85.1% of the respondents were informed about the Asian tiger mosquito by media and internet (18). Surprisingly, information leading to awareness through health care workers seem to be rare. Two studies found only 8–9% of the respondents were informed about mosquitoes and MBIDs by physicians (28, 38). This contrasts with endemic countries where between approximately 50 to 80% of the respondents are aware by health professionals that seems to play an important role in prevention about MBIDs and mosquitoes (39, 40).

#### 3.1.2 Attitudes toward nuisance and health risk

Studies on MBIDs and nuisances caused by mosquito bites have shown that most respondents are more concerned about the negative impact on their well-being than the related health risks. In the reviewed papers, between 44 and 80% of the respondents considered mosquito bites to be a serious nuisance during their activity periods (14, 18, 19, 21, 41, 42). For instance, Davidson (14) found that in St Johns County (Florida, USA) 80% of the respondents were bothered daily or several days per week (14). In Lyon (France) the survey amongst people participating in community gardens showed that the majority (81.3%) of the respondents were highly or moderately concerned by the presence of mosquitoes, with most of them being more preoccupied by nuisance and impacts on their quality of life than by disease transmission (18). An Italian study compared the attitudes of three groups of people: the native population and Italians originating from two endemic regions of India (21). In the group of native Italians 58.3% of the respondents were disturbed by mosquito bites whereas only 0.6 and 14.3% of the Malayalis and Punjabis, respectively, stated that they were concerned with mosquito bites. Some papers show that nuisance due to mosquito bites leads to changing activities, mostly by reducing time outdoors. For instance, in Washington, a large majority of respondents (61%) reported a change in activities (41).

Among other studies that have focused on the health impact of MBIDs and mosquito bites, some were conducted in the south of the USA, where MBIDs are present without being endemic. These studies demonstrated a great variety of risk perception according to the type of groups investigated. For instance, 95.5% of surveyed households in New Orleans stated that mosquitoes represented a major risk of transmitting diseases (37). Curry et al. (25) found that almost one woman out of three interviewed in the gynecology department was concerned about the spread of ZIKV, particularly when pregnant. This concern was amplified among women who had given birth at least once (82.6%) (25). In contrast, only 23% of surveyed workers in Miami said they were moderately or extremely concerned about contracting a disease transmitted by mosquitoes (42). Similarly, respondents in South Texas had, on average, moderate fear of mosquitoes and the diseases they transmit, although they perceived MBIDs as a serious problem (17). In Greece, Kolimenaskis et al. (2022) showed that 83% of the respondents consider mosquito as a problem for nuisance and health (23).

In contrast to the southern United States, studies conducted in Europe showed relatively lower levels of concern regarding mosquito-borne infectious diseases (MBIDs). In France, vulnerability to MBIDs was found to be low or moderate depending on the populations studied (ranging from 21.7 to 53.1% for random respondents in south of France and 27.7% for gardeners of community gardens) (18, 31). In the Lazio region (Italy), 51% of respondents reported that they felt never or rarely worried about *Aedes*-transmitted diseases compared to 29% who considered themselves very or extremely worried (21). Caputo et al. (21) found a large difference in attitudes between the investigated categories. Only 15.2% of native Italians reported being worried about diseases transmitted by *Ae. albopictus*, while among Malayali and Punjabi respondents, the proportions were 28.8 and 26.7%, respectively (21).

Overall, these results show that despite concern about MBIDs in nonendemic regions, perceived risks of epidemic-prone diseases are generally rather moderate, and seem to be related mainly to the characteristics of the surveyed group.

#### 3.1.3 Mosquito mitigation practices and personal protection

The respondents in the analyzed surveys adopted different practices to avoid mosquito proliferation or to protect themselves against mosquitoes. Regarding these practices, questions in KAP studies addressed the use of personal protective measures and the management of potential breeding sites.

The majority of respondents in southern Texas (80.5%) declared that they correctly eliminated standing water (17). In a Spanish survey, the researchers observed that 76% of local population in Saint Cugat del Vallès, north of Barcelona, managed standing water to avoid potential breeding sites (13). Richards et al. (43)reported that 67% of respondents in north Carolina reported removing empty containers such as flowerpots, birdbaths or tires (43). In France, 82.1% of gardeners in community gardens responded that they actively fought against mosquitoes by eliminating standing water containers (18). Despite the efficiency of this mechanical method, some surveys show that it is not systematically used by local residents. Only 40% of respondents in southwest Virginia and 50% in New York reported regularly or occasionally removing standing water containers (12, 34). A survey conducted in southern France found that only 17.7% of respondents eliminated standing water and approximately one out of two stated that they did not follow any of the practices recommended by public health authorities (27). Even though construction workers are considered a population at risk for many MBIDs, 80% of surveyed construction industry employees in Miami did not use preventive measures such as removing standing water (42, 44).

Several studies also analyzed personal protective measures against mosquitoes. Although personal protective measures are not intended to prevent mosquito proliferation, they contribute to limiting the number of bites and thus slowing the propagation of MBIDs. Personal protection takes many forms such as the use of repellents, wearing covering clothing, using door or bed nets, and limiting outdoor activities especially at hours when mosquitoes are active. In general, the use of repellents was the most frequently cited method by KAP respondents, probably because they are easy to apply and decrease contact between mosquitoes and humans. Overall, between 60 and 90% of the respondents reported using repellents to protect themselves (18, 28, 37, 38, 45). However, in the study conducted by Caputo et al. (21), the majority of surveyed Italians (53%) reported that they used nothing to avoid *Aedes* bites, only 23% used environmental repellents at home, and 15% used personal repellents (21). Some studies have shown that bed nets or covering clothes that offer the advantage of being environmentally friendly and have no impact on human health are commonly used for personal protection (18, 22).

### 3.2 Explanations of urban citizens’ practices in the eradication of breeding sites

#### 3.2.1 Influence of knowledge and concern about mosquitoes and MBIDs on mosquito control practices

People’s level of knowledge and concern about mosquitoes and MBIDs clearly have an impact on their control practices according to the previous publications that have addressed this issue (10 of the reviewed articles). Some KAP surveys show that a high level of knowledge, for example, of the mosquito life cycle is essential to identify potential larval habitats and implement more appropriate practices (17, 18, 21, 27, 41, 46). Caputo et al. (21) found that a low level of Italian citizens’ knowledge (21% of correct answers) was associated with a low level of practices to reduce or remove breeding sites (0.7%) (21). Raude et al. (27) found in their study in French Mediterranean regions that a high ability to identify the *Aedes* species was highly associated with protective behavior by the respondents (*p* < 0.001) and resulted in the elimination of standing water (27). In Washington (USA), Dowling et al. (41) demonstrated that approximately half of surveyed respondents who recognized breeding sites reported that they removed standing water (41). To our knowledge, only two studies have shown an opposite effect. Duval et al. (18) revealed no significant relationship between the level of knowledge and type of actions taken against mosquitoes and their breeding sites (18). In this survey focused on the community gardeners in Lyon (France), attitudes, more than knowledge, appear to be an explanatory factor. Gardeners who were very concerned about mosquitoes were overrepresented in the group of gardeners who modified all or some practices because of mosquitoes (76 and 62% of the respondents, respectively). Only 27.9% of respondents who had not changed their habits in the garden due to the presence of mosquitoes were concerned about the presence of mosquitoes. This finding suggests that the more concerned community gardeners are about the presence of mosquitoes in their gardens, the more likely they are to take action against this species (18). Moise et al. (37) observed in New Orleans (USA) that 20.5% of respondents had good practices regarding mosquito control despite a lack of knowledge about potential breeding sites (37).

#### 3.2.2 Impact of socioeconomic status on mosquito management practices

Most studies show that knowledge and practices depend on the socioeconomic status of the respondents, such as employment status, educational level or income (12, 17, 27, 28, 37, 41, 47, 48).

Regarding household income, Walker et al. (48) found that the odds of finding containers colonized by *Ae. aegypti* in outdoor premises were positively associated with low-income households in Arizona (United States) (48). Moise et al. (37) observed that 31.6% of the New Orleans respondents in high-income households had negative practices against mosquito bites compared to 35.2% of respondents from low-income households. They also showed that employed individuals had significantly higher knowledge regarding mosquito control and practices to prevent mosquito proliferation and mosquito bites than unemployed individuals (OR: 1.00; 95% CI [0.000–0.006]) (37).

Educational level seems to be another key factor that explains good mosquito knowledge and appropriate practices (12, 17, 27, 28, 41, 47, 49). In Washington, respondents without a college degree had significantly more containers that could receive rainwater at their homes than respondents with a college degree (41). However, several studies found no link between the socioeconomic status of respondents and their level of knowledge and practices regarding mosquitoes and MBIDs (18, 19, 31, 34, 37). In a study of nonprofessional gardeners in France, Duval et al. (18) found no correlation between knowledge, practices and educational level (18). The absence of a significant relationship is probably because the respondents in the survey were all participating in community gardens, where awareness of mosquitoes is generally high. An American study in southwestern Virginia concluded that education and income were not significant predictors of the level of knowledge and practice (34). This study focused on the population of central Appalachia, which is an economically deprived region where financial resources for public health are low. In a study in Germany, educational level did not appear to have a significant effect on knowledge of *Anopheles* breeding sites (19). This could be explained by the fact that 54% of respondents claimed to have no mosquito breeding habitats close to their homes or had no knowledge of them. Following these examples, the lack of relationship between education and income with level of knowledge and practices could be the result of lower education levels in the target population.

Despite the lack of correlation observed in some studies, it seems that the socioeconomic level of residents explains their level of knowledge and behavior in regard to mosquitoes and their potential breeding sites. Numerous publications show that a high living standard and educational level are related to more efficient management of potential larval habitats and reduced frequency of breeding sites in a neighborhood. Similar observations are found in endemic countries (50–52).

#### 3.2.3 Role of demographic factors on protective behavior against mosquitoes

In addition to socioeconomic characteristics, several studies have investigated the effect of demographic variables on knowledge and practices regarding mosquitoes (12–14, 17, 19, 21, 22, 27, 38, 43, 53–55). Gender appears to be a key factor explaining the knowledge and behavior of respondents (12, 27, 31, 34, 43, 49). Overall, with regard to mosquitoes and MBIDs, female respondents show better knowledge and more appropriate practices. Studies in France and China revealed that the percentage of women who adopted protective behavior against mosquito bites (i.e., the use of chemical repellents or covering clothes) was significantly higher than male respondents (27, 31). A survey conducted in New York demonstrated that women had overall better knowledge about WNV than men (12). Additionally, in south-western Virginia, women declared themselves to be better informed about MBIDs and more concerned about health risks linked with mosquito bites and contracted diseases than men (34). Higher knowledge and concern in women could be explained by the fact that women are more anxious about health problematics and, in general, exhibit more responsibility than men regarding ‘care’ (56–59). A few publications have reported gender differences in practices regarding mosquito management. For example, two studies revealed that women respondents declared that they adopted better management of breeding sites than men, such as removing standing water and outdoor measures (43, 49).

Several studies have revealed that age also influences knowledge and practices regarding mosquitoes. Although the age categories are not similar among studies, most studies have found that older respondents had better knowledge and practices regarding the management of breeding sites (12, 41, 47, 53). For instance, individuals aged 45+ years in Washington reported a better level of mosquito ecology knowledge and were more active regarding standing water management (41). Similarly, Maeda et al. (47) found that self-reported source reduction was more common for respondents over 50 years than for younger participants in Washington D.C. and Maryland (USA) (47). In Key West and Tucson (USA), respondents aged 35+ years reported that they cleared standing water in their property more often than younger groups (53). Different practices according to age groups could be explained by the fact that older persons are more likely to garden than younger residents, and thus are more familiar with mosquito larval habitats.

In regards to protective behavior, a few studies have revealed opposite results (31, 34, 49). Among respondents living in Mediterranean France, Constant et al. (31) observed that older individuals (≥68 years) were negatively correlated with personal protection against mosquitoes (Odd Ratio = 0.34) (31). However, this result should be interpreted with caution because, most of these respondents were retired which could influence specific mosquito protective behavior. In China, younger age was found to be a good predictor of adopting effective outdoor protective measures (49).

### 3.3 Knowledge and attitudes regarding mosquito control methods

#### 3.3.1 Knowledge of mosquito control

Many types of mosquito control methods exist including physical, chemical, biological, mechanical and environmental methods. Mosquito control implies the local residents have good knowledge of the mosquito life cycle and be aware of their role in prevention. We found only three studies that examined people’s knowledge of mosquito control in nonendemic countries (6, 18, 55). Claeys et al. (9) investigated social knowledge and perception of mosquitoes and mosquito control methods in France regarding larvicide spraying in wetland (6). This study is based on field surveys conducted between 1995 and 2006 in the Rhone river delta (South of France) and in some Alpine valleys (East of France). In total, the population sample of 639 persons were interrogated via standardized questionnaires or semi-directive interviews. The results showed that in the delta of the Rhone River in France recurring campaigns of larvicide applications were poorly known by the local population (6). More than 40% of the respondents did not know who was responsible for the mosquito control campaigns in the region, although mosquito control has been controversial for more than 10 years. Another French survey in Lyon investigated the level of knowledge about biological control methods among nonprofessional gardeners in community gardens (18). It is interesting to highlight that only 20.5% of the respondents had ever heard about biological control methods. Among them, more than one-third were unable to describe the concept of biological control. Interestingly, only 3.4% of the gardeners were able to explain different biological methods like predation, use of *Bt* toxin, and 2.3% confused these methods with mechanical methods. Despite a general lack of knowledge, the gardeners wanted more information about alternative control methods, as revealed by the average score of 4.16 on a five-point Likert scale on this issue. This study also found that respondents who had already heard about biological control methods also had a better practice regarding mosquito management. Another study on public opinion regarding mosquito control in four locations in Florida, USA, showed low knowledge of mosquito control methods (26, 55). The respondents pointed a lack of notifications prior to spraying campaigns and declared they would like to know more on this control method. To our knowledge, no study has yet investigated participants’ knowledge of the sterile insect technique (SIT) or the use of the bacterium *Wolbachia* which are both implemented in endemic areas (60). SIT consists in releasing sterilized males in a target area. The systematic and repeated release of unfertile males decreases mosquito population over time (60). Releasing mosquitoes with *Wolbachia* over several months can reduce the number of a specific mosquito species, such as *Ae. aegypti* or compete with viruses within mosquitoes (61).

Knowledge questions in KAP surveys are largely restricted to mosquitoes and MBIDs. To increase the implications for the local population of mosquito control in nonendemic countries, there is a need for both better information on existing control methods and more surveys dealing with this issue.

#### 3.3.2 Attitudes toward mosquito control methods

Attitudes toward mosquito control methods have been addressed in a larger number of surveys than the level of knowledge regarding those methods (6, 15, 22, 27, 29, 45, 55, 62). Understanding the population’s perception of mosquito control and its effectiveness is necessary to assess which practices, e.g., protective behaviors or actions against mosquito proliferation would potentially be well accepted and used by the population. Claeys et al. (9) found two extreme perceptions in two regions of France, with one part of the population in favor of mosquito control regardless of the method and the other part against *Bti* larvicide spreading (6). For instance, in Camargue, 82% of the respondents were favorable to mosquito control. Additionally, the majority of the respondents living in areas where mosquito control had been implemented for 10 years would not want to return to a situation without mosquito control interventions. However, even the respondents who were the most favorable to mosquito control had some reservations; nearly 60% of all respondents underlined the potential damage to the ecosystem (6, 63). A Greek study analyzed residents’ perception of SIT and highlighted high acceptance by most residents (22). Among the participants, 92.5% thought that SIT was a good idea and on an effectiveness scale of 0 to 5, 70% answered between 4 and 5. In addition, 79.5% of the respondents considered that this method had more advantages than chemical methods. They also expressed concerns regarding the chemicals used and their potential environmental and human health impacts. Moreover, they stated having used most of the recommended at-home mosquito control mechanical methods such as keeping doors and windows shut and eliminating standing water reservoirs (55).

Overall, it is clearly important to understand the public perception and beliefs about mosquito control. Another question that is rarely investigated in the KAP surveys and should be further developed is the perception of individuals’ role in mosquito control. To the best of our knowledge, only one KAP survey has analyzed the perception of 126 respondents impact of their own mosquito avoidance behavior may have on mosquito proliferation in their home and neighborhoods (15). More than half of the respondents (53%) considered that removing standing water from containers and flowerpots, have some impact on the local mosquito population. Only 14% thought that their action had a very low impact, while 33% believed their impact very significant around their home and neighborhoods. Convincing people of the impact of their actions represent an educational opportunity to motivate population to contribute to mosquito control.

#### 3.3.3 Attitudes toward awareness plans and effectiveness of citizen sciences

Another important factor that improves effectiveness of control and prevention measures is the willingness of the population to adapt their practices and routines. Awareness plans, information and education are necessary as a complement to vector control campaigns in order to limit mosquito proliferation thus vector-borne diseases. In Washington DC, scientists’ interventions to control mosquitoes have significantly reduced the number of breeding sites (41). According to a Greek study, raising awareness through printed educational materials alone has had no effect (20) or the opposite effect due to a decrease of MBID concern after the prevention campaign (64). Several studies highlighted that respondents perceived educational efforts by local authorities too low and asked for better strategies to enhance awareness about mosquito and MBIDs. For instance, Rampold et al. (55) revealed that all Florida respondents agreed that education regarding mosquito control efforts and their impact was needed (18, 55). Several studies also revealed that respondents identified local governments or public health organisms as responsible for this health issue (15, 18, 37). Despite this observation, people concerned with mosquitoes in their neighborhood may have other information channels. For instance, 73.2% of the nonprofessional gardeners in community gardens conducted their own research for information on Internet (18). The opposite was observed in Florida where most of the respondents had not research actively regarding mosquito control (55).

Citizen science is currently considered as a main driver to motivate and involve the public as citizen scientists. Volunteers are commonly involved in data collection, but can also be involved in initiating questions, designing projects, disseminating results, and interpreting data (UNEP 2019). With the aim to facilitate the public participation and collaboration in scientific research to increase scientific knowledge, citizen science could help for mosquito surveillance and mapping. Heym et al. (19) found that respondents that were involved in a citizen science project were able to recognize potential larval habitats and modified their practices regarding breeding site management (19). Some citizen science projects have already shown their effectiveness such as the “Mosquito Alert” project that aims to make an inventory, via the use of a smartphone app by volunteers, of places where of various mosquito species in Europe are observed. After validation and correction by entomologists, the collected information is used to create early alert for MBIDs risk through predictive models (65, 66).

## 4 Conclusion

**Figure 1:**
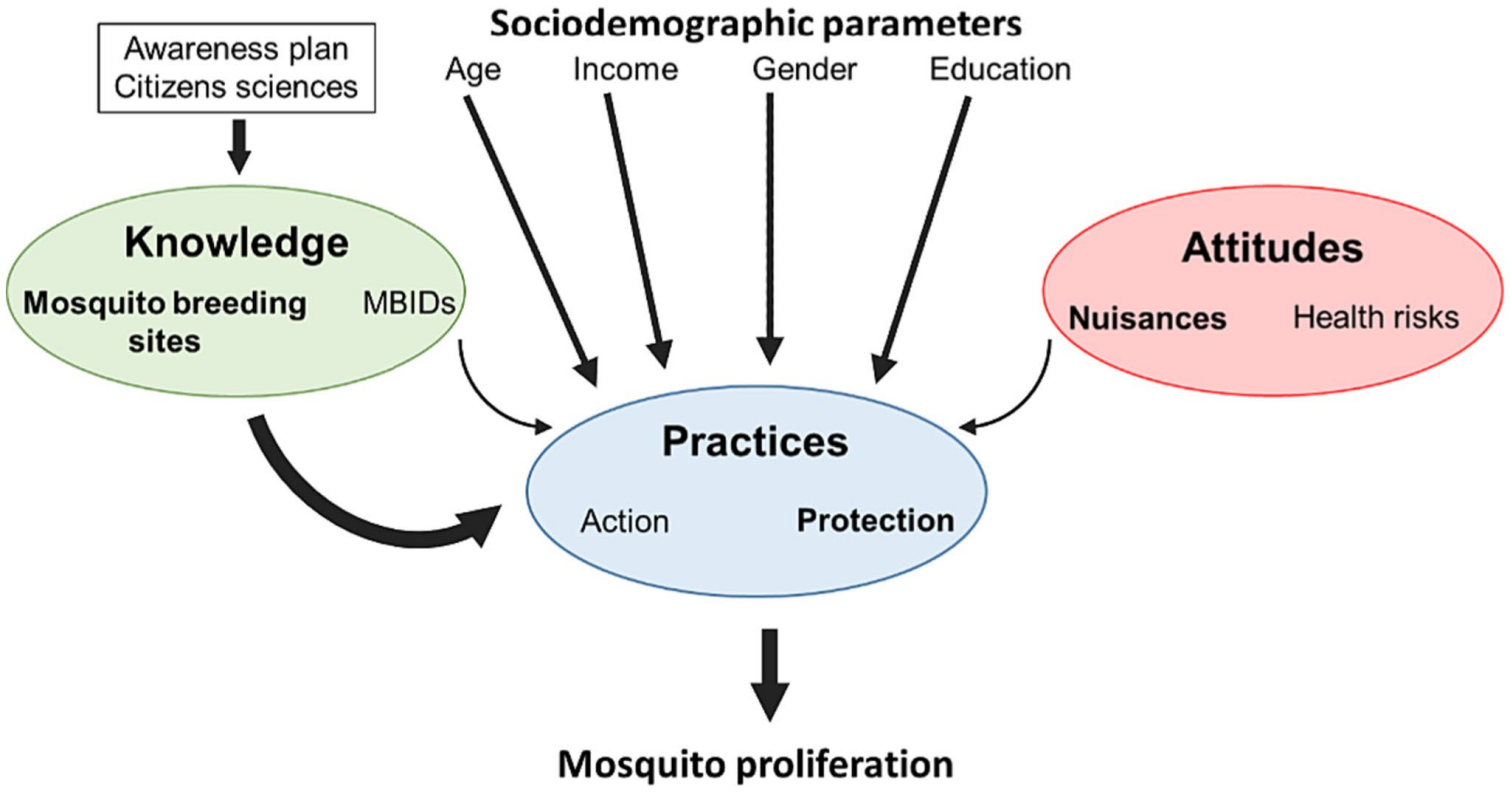
Impact of knowledge, attitudes and sociodemographic parameters on mosquito control practices and dispersal in nonendemic countries. Regarding knowledge, recognizing potential breeding sites and being aware of MBIDs influence the level of mosquito practices. Citizen sciences seems to be a high awareness plan to increase the knowledge of participants. Regarding attitudes, respondents perceived mosquitoes more as a nuisance than a vector of diseases. Regarding sociodemographic characteristics, women, older people and people with higher incomes and higher levels of education were positively associated with better practices. Regarding practices, respondents used personal protection, especially mosquito repellents, to protect themselves from mosquito bites, and they also took action against mosquitoes by eliminating potential larval habitats. The degree of effect is proportional to the width of the arrow.

This literature review sheds light on the disparities in knowledge, attitudes, and practices pertaining to mosquitoes and MBIDs in nonendemic regions. The understanding and behavior of the local populations have an impact on the proliferation of mosquitoes within urban areas. KAP questionnaires are commonly employed to evaluate the factors influencing both positive and negative behaviors, as well as the knowledge and attitudes concerning MBIDs. These surveys play a crucial role in identifying undesirable behaviors within the population and pinpointing gaps in awareness regarding diseases and vectors. Furthermore, they aid in the development of effective strategies that engage the community in vector control efforts. This review focused in particular on factors explaining what influences people’s practices regarding mosquitoes, as raising awareness about preventive measures and protective behaviors is of primary importance to face the risks of vector-borne diseases. Different explanations were put forward in the surveys. Knowledge about mosquito breeding sites and awareness of mosquito’s role in some MIBDs are key factors that explain more or less appropriate mosquito control practices on the individual level. Moreover, socioeconomic and demographic status also explain the variability of observed practices (Figure 1). The reviewed studies revealed that a significant portion of survey participants possessed only partial knowledge of potential mosquito breeding sites. This is an information of utmost importance, as a very good knowledge of these breeding sites is crucial for individuals to adopt effective practices aimed at preventing the spread of mosquitoes. Another important result that we found in these studies is that there are varying levels of awareness and understanding regarding the infectious diseases transmitted by mosquitoes based on geographical locations. These variations are clearly dependent on the frequency and the scale of the sporadic outbreaks of MBIDs in the surveyed regions. Surveys conducted in continents with frequent outbreaks resulting from local transmissions of MBIDs, such as the Southern United States, consistently demonstrated a high level of knowledge. In contrast, in Europe, where MBIDs remain sporadic, and epidemic occurrences are largely reliant on imported cases, awareness regarding health risks associated with mosquitoes tended to be less widespread. Alongside these results on knowledge, several studies have also shown that most respondents in the surveys are more concerned about the negative impacts on their well-being than the related health risks. Despite a certain knowledge about MBIDs, the perceived risks of epidemic-prone diseases are generally rather moderate in nonendemic regions. We also noticed that multiple studies indicate that a strong understanding of potential mosquito larval habitats and heightened awareness of mosquito presence and MBIDs have a positive impact on the practices. Individuals who are both concerned and well-informed tend to adopt more appropriate measures in combating mosquitoes. There also seems to be a gender disparity, with female respondents that in general are better informed about mosquitoes and MBIDs, which tend to lead to the adoption of more effective practices against mosquito proliferation. Many studies within this review analyze how knowledge and practices are influenced by the socioeconomic status of respondents, and the results show in particular that educational level seems to be a key factor that explains good mosquito knowledge and appropriate practices. This review has also highlighted a scarcity of KAP studies focusing on the population’s awareness and perception of different kinds of mosquito control methods and their efficiency. To enhance local community engagement in mosquito control efforts in nonendemic regions, there is a pressing need for improved dissemination of information regarding existing control methods (both individual and control methods used by local authorities), coupled with an increase of the number of surveys addressing this topic. It is evident that further evaluation is necessary to identify sustainable or eco-friendly practices or measures against mosquito proliferation that could be readily embraced and adopted by the population.

All these findings show the crucial role of knowledge in promoting good practices, emphasizing the necessity for widespread awareness and information campaigns. Several studies indicated that respondents perceived local authorities’ educational initiatives as insufficient and expressed the need for more effective strategies to enhance awareness regarding mosquitoes and MBIDs. However, there is uncertainty surrounding the definition of an effective awareness and information campaign, when it comes to content, medium and distribution method. Some of the reviewed studies revealed that under specific circumstances, informational and educational efforts may fail to achieve their intended outcomes and, in some cases, could even lead to unintended adverse effects on attitudes and practices concerning mosquitoes and MBIDs. Consequently, there is a great need for additional surveys that evaluate the impact of educational initiatives, information dissemination, and awareness campaigns.

Given the importance of the emerging health risk in nonendemic countries and regions due to mosquito proliferation, and in particular the tiger mosquito, this review of studies based on KAP surveys provide decision-makers with new information to better manage mosquito spreading and mosquito-borne diseases.

## Author contributions

PD wrote the first draft of the manuscript. CA-L and CVM reviewed and edited the manuscript. All authors contributed to the article and approved the submitted version.
